# Multimodality hard-x-ray imaging of a chromosome with nanoscale spatial resolution

**DOI:** 10.1038/srep20112

**Published:** 2016-02-05

**Authors:** Hanfei Yan, Evgeny Nazaretski, Kenneth Lauer, Xiaojing Huang, Ulrich Wagner, Christoph Rau, Mohammed Yusuf, Ian Robinson, Sebastian Kalbfleisch, Li Li, Nathalie Bouet, Juan Zhou, Ray Conley, Yong S. Chu

**Affiliations:** 1 National Synchrotron Light Source II, Brookhaven National Laboratory, Upton, NY 11973, USA; 2 Diamond Light Source Ltd, Didcot, Oxfordshire, OX11 0DE, UK; 3 London Centre for Nanotechnology, University College London, London, WC1H 0AH, UK; 4 Research Complex at Harwell, Rutherford Appleton Laboratory, Didcot, OX11 0FA, UK; 5 Advanced Photon Source, Argonne National Laboratory, Argonne, Illinois 60439, USA

## Abstract

We developed a scanning hard x-ray microscope using a new class of x-ray nano-focusing optic called a multilayer Laue lens and imaged a chromosome with nanoscale spatial resolution. The combination of the hard x-ray’s superior penetration power, high sensitivity to elemental composition, high spatial-resolution and quantitative analysis creates a unique tool with capabilities that other microscopy techniques cannot provide. Using this microscope, we simultaneously obtained absorption-, phase-, and fluorescence-contrast images of Pt-stained human chromosome samples. The high spatial-resolution of the microscope and its multi-modality imaging capabilities enabled us to observe the internal ultra-structures of a thick chromosome without sectioning it.

Understanding the complex hierarchical architecture of chromosomes remains a central topic in biology because it plays an important role in determining the mitotic process of cell division. A variety of microscopy techniques have been employed to image chromosome structures, but each has its own advantages and limitations. The resolution of the light microscope is limited to about 250 nm and therefore, does not provide information relating to the finer internal structure^1^. More powerful fluorescence microscopy methods such as 2D super-resolution imaging by photoactivated localization microscopy (PALM) has been used but is limited to the dyes used^2^. Other imaging methods such as scanning electron microscopy (SEM) only visualizes the surface of the sample^3^. Transmission electron microscopy (TEM) has provided high resolution, but it is limited to thin samples and cannot be used reliably to image the micron-sized chromosomes because the electrons cannot penetrate through the sample^4^,^5^. TEM serial sectioning has been used after the sample has been embedded into resin but it is labor-intensive, time-consuming since the image sections have to be handled manually^6^,^7^. Recently Focused ion beam SEM (FIB SEM)^8^ and serial block face SEM (SBFSEM)^9^ have been used to image internal structure of chromosomes, however involve sectioning. Intact chromosomes have been imaged in a pioneering X-ray study using 3D X-ray diffraction imaging^10^. X-rays have the advantage over other methods that they have a shorter wavelength and can penetrate through the entire sample where no sectioning is needed. In recent years, hard x-ray microscopy emerged as a promising technique for imaging thick biological samples due to the excellent penetration power of hard x-rays that electron microscopy cannot offer, a modest- to high-spatial resolution that optical microscopy cannot achieve, and high detection sensitivity to trace metals. Scanning^11^,^12^,^13^, full-field^14^ and lensless^10^,^15^ x-ray microscopy techniques have been developed, with a spatial resolution ranging from around 25 nm to 100 nm. Scanning hard x-ray microscopy, because of its multi-modality imaging capabilities for structural- and elemental-mapping, attracts much interest for biology applications. In this technique, we employ a nanofocusing optic to focus the hard x-rays down to a small spot and then the sample is raster-scanned while surrounding detectors collect all signals generated at each scanned position to form images simultaneously via different contrast mechanisms. As a result, its resolution is limited by the size of the nanobeam. Due to the extreme difficulties associated with fabricating hard x-ray nano-focusing optics, the best scanning resolution in the hard x-ray regime reported to date for biological studies is around 30 nm, achieved using a Fresnel zone plate (FZP)^13^. This resolution still is not sufficient to resolve ultrastructures in many biological samples, for example, the high-order structure of mitotic chromosomes.

We developed a scanning hard-x-ray microscope based on the multilayer Laue lens (MLL)^16^, a new class of focusing optic that exhibits superior performance in the hard x-ray regime^17^. An MLL resembles a one-dimensional FZP, but has its own characteristics due to the strong dynamical diffraction effects. In theory, an MLL is capable of sub-nanometer focusing with more than 60% efficiency^18^. To date, MLL optics have produced an 11-nm line focus^19^ and have been used for several scientific applications^20^,^21^. When the resolution of x-ray imaging is reduced down to a single digit, the long-standing dream of imaging the structure of DNA directly in real space with x-rays may be realized. Taking advantage of the high spatial-resolution and multimodal-imaging capabilities offered by the MLL microscope, we discuss here our quantitative x-ray imaging of a chromosome with nanoscale resolution using simultaneously acquired absorption-, phase- and fluorescence-contrast imaging methods.

## Results

### Experimental details

The scanning microscope we used was equipped with two MLLs, one with 43-µm aperture and a 4 nm-wide outermost zone and a focal length of 4.2 mm at 11.8 keV; the other with a 53-µm aperture, a 4 nm-wide outermost zone and a focal length of 5.2 mm at 11.8 keV. Both optics were first aligned with respect to the incident beam and then aligned with respect to each other to produce a two-dimensionally focused spot at the same plane. Eight degrees of freedom are available to fully align the optics, implemented with a compact design and high-stiffness piezo-based stages. The recorded overall drift of the system was less than 2 nm/hour, which we measured independently on a reference sample. A detailed description and performance characterization of the instrument is published elsewhere^16^. Because the focus was separated from the direct beam by a few microns and the chromosome sample was small and well isolated, we intentionally performed this study without using an order-sorting-aperture (OSA) in order to increase the working distance^22^.

**Figure 1:**
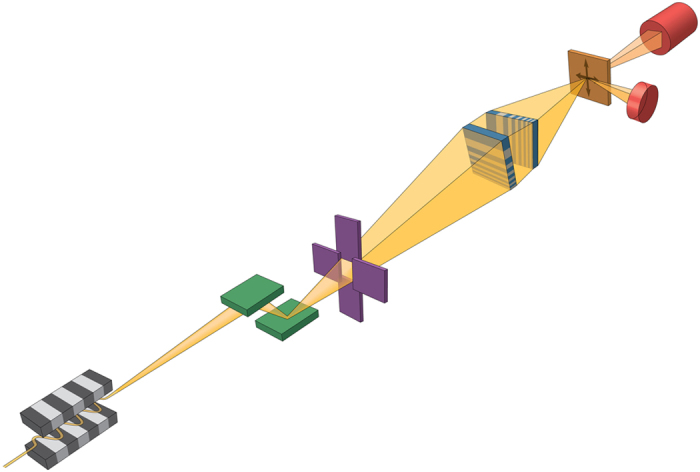
Schematic of the experimental setup. A bright and coherent x-ray beam is generated from an undulator source. A monochromatic beam is then selected by the double-crystal monochromator and further shaped by a set of slits. The x-ray beam is focused down to a 13 × 33 nm^2^ spot by two MLLs placed in a cross geometry. In this study because the sample was well isolated, an order-sorting-aperture (OSA) was not used to maximize the working distance^22^. A far-field pixel-array detector and an energy-dispersive detector are used to collect different types of signals as the sample is raster scanned through the focus, thereby yielding absorption-, phase- and fluorescence-contrast images simultaneously.

The experiment was conducted at the 250 m long imaging and coherence beamline I13L of the Diamond Light Source, UK. A monochromatic x-ray beam at 11.8 keV was selected by a Si (111) monochromator, and then was focused down to a nanobeam of ~13 × 33 nm^2^ (also see supplementary materials), with a flux of 1.3 × 10^7^ photon/s in the focus. The absorption in the sample is a few percent, which, at first glance, yields a total radiation dose of ~10^8^ Gy, assuming an average dry mass of 84 × 10^−12^ g for a chromosome^23^. This dose is an order of magnitude larger than that reported previously in soft x-ray transmission microscopy^24^. A more detailed estimate reveals that the actual dose is much lower. As will be discussed in detail later, our sample was heavily stained with Ag which absorbed most x-ray photons. When its mass is taken into account, the actual dose is estimated to be ~4 × 10^5^ Gy. No radiation damage was detected by comparing two successive scans, and this agrees with the previous observation^24^. The chromosome sample was positioned at the focal plane, and was raster-scanned in two lateral directions, as shown in Fig. 1. Different types of signals at each scanned position were collected by two detectors placed around the sample. The excited x-ray fluorescence photons were analyzed by an energy-dispersive detector (AXAS-M, KETEK GmbH) positioned at about 90 degree with respect to the beam’s direction to give element-specific information. A pixel-array detector (Timepix, 55 μm, 512 × 512 pixels) was used to capture the transmitted beam’s signal in the far-field, about 1.4 m downstream from the sample. We then analyzed the recorded transmission image at each scanned position by a non-linear fitting algorithm to produce both the absorption-contrast and phase-gradient images of the sample^21^. After the scan was complete, the phase image was reconstructed from the measured phase gradients using a Fourier integration method^21^.

### Imaging results

**Figure 2:**
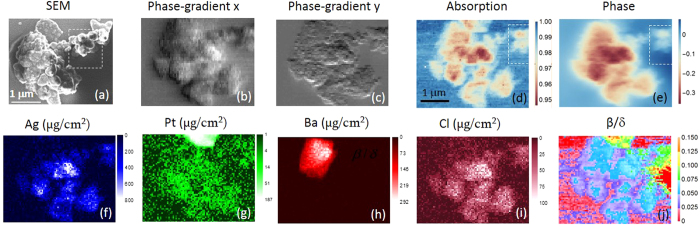
(**a**) scanning electron micrograph (SEM) of the examined chromosome (performed after the x-ray measurement). Many globules can be seen on the surface. (**b**,**c**) are the x-ray phase-gradient images in horizontal and vertical directions, respectively. They provide enhanced contrast to small features. (**d**) is the transmission image showing the absorption contrast. Maximum absorption is about 5%, seen around the center of the image. (**e**) is the phase image, reconstructed from (**b**,**c**). Maximum phase retard is seen around the same position in (**d**) where a high concentration of Ag (**f**) is present. (**f**–**i**) are the concentration maps of Ag, Pt, Ba and Cl, respectively. The units are μg/cm^2^. Note the measured concentration is a projection through the entire thickness. In order to show the weak signal in (**g**), log scale is used. (**j**) depicts the *β*/*δ* ratio variation. It indicates the composition change. The Pt particle along the top edge can be seen clearly in this map. The scan-step size in (**b**–**j**) is 50 nm.

The sample consists of purified chromosomes prepared from b-lymphocyte cells from a Yoruba cell-line (GM18507) at passage 4 and prepared according to the protocol described by Shemilt *et al.*^3^ and Yusuf *et al.*^9^. It was stained with 5 mM platinum blue by placing a drop on top of pre-dried chromosomes for 30 minutes, following standard procedures^25^. Silver sulfate (Ag_2_SO_4_) was used as a precursor in the preparation, and Ag’s concentration was expected to be very low. Previously it was found that the Pt binds primarily to the DNA^26^. Therefore, the distribution of Pt can be used to map the DNA structures. The sample first was examined under a scanning electron microscope (SEM). Figure 2a is the SEM image, showing the sample’s morphology; its thickness was estimated to be between 1–2 µm. Due to the shallow depth of penetration by electrons and the relatively large thickness of the sample, the internal structure, which is of the most interest, cannot be viewed with SEM. In contrast, hard x-rays can penetrate through a thick sample (at 11.8 keV, the penetration depth for biological samples is over 3 mm), providing a non-invasive imaging method for structural characterization.

Figure 2b–i are, respectively, the phase-gradient, absorption-contrast, phase-contrast and Ag-, Pt-, Ba-, and Cl-fluorescence images of the chromosome acquired simultaneously with a single 2D raster imaging. The absorption contrast mostly reflects the concentration of heavy metal stains in this case, since the organic material almost is transparent at 11.8 keV. From the absorption, we can calculate the attenuation factor that is proportional to the sample thickness, *t*. Phase-gradient images describe the phase change of the emerging wavefront over a unit length in two orthogonal directions and are very sensitive to thickness and compositional variations within the sample. Thus, they enhance the contrast of small features. The phase image is obtained by integrating the phase gradients afterward^21^. The measured phase and the attenuation factor respectively are the projections of the real- and imaginary-components of the complex refractive index through the thickness of the sample and are functions of its composition and the thickness (see methods). Hence, their ratio, which cancels the unknown thickness, leads to a novel type of contrast for composition (Fig. 2j) that has been successfully applied to studies of different type of samples before^21^,^27^,^28^,^29^. Ag came from the precursor used in the preparation process of the platinum blue stain^30^ and deposits have been previously seen^31^ (see methods). Its high concentration is due to an incomplete rinse during the preparation and is not desired. Ag binds preferentially to protein. The strong Ba signal shown in Fig. 2h corresponds to an unintended contamination particle introduced into the sample. There were BaTiO_3_ samples prepared about the same time, so there was a change of cross contamination. Ti signal was also detected in the same area (not shown), confirming that it was a BaTiO_3_ particle. This particle is not apparent in the SEM image, but is clearly seen in the x-ray image, owing to x-ray’s superb penetration power and high sensitivity for detecting metals. Chlorine exists at multiple stages of the preparation process. The distribution of Pt is of the most interest, since it binds to DNA. A large clump of Pt is seen on the top, which results from excessive Pt accumulating along the edge of the sample as the stain evaporates. A low density of Pt is observed inside the sample, with its distribution well correlated to the sample morphology. In Fig. 2d,e, the maximum absorption and the largest retarded phase occur around the center. As discussed earlier, the absorption- and phase-contrast images respectively represent the variations in the thickness and composition. Figure 2j is a composition map, which eliminates the effect of variations in thickness. For Pt, Ag, BaTiO_3_, Cl and protein/DNA, their *β*/*δ* ratios at 11.8 keV are, 0.13, 0.05, 0.06, 0.02 and 0, respectively. In other words, the higher density of the material is, the greater value of its *β*/*δ* ratio will be. In this map, the Pt particle on the top can be seen well. The BaTiO_3_ particle, however, is not clearly discernable from Ag because their *β*/*δ* ratios are close. In general, the composition map shows a good correlation with Ag, but not with Pt (except around the upper boundary where the Pt particle is located). Therefore, the dense area shown in Fig. 2j mostly corresponds to a conglomeration of proteins, but not of DNA.

**Figure 3:**
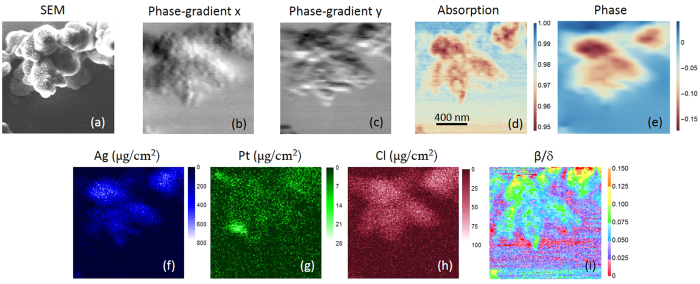
Magnified x-ray images of the enclosed area in Fig. 2a,d,e. (**a**) is the SEM image. (**b**,**c**) are phase-gradient images in horizontal and vertical directions, respectively. Small globules can be seen. (**b**) shows more fine features than (**c**), indicating a better resolution in the horizontal direction. (**d**) is the absorption-contrast image. Fibrous ultrastructures can be observed. (**e**) is the phase image (unit in radian). Globules are not as apparent as in (**b**,**c**) due to the weak contrast and worse resolution. (**f**–**h**) are the distributions of Ag, Pt and Cl, respectively. Units are μg/cm2. (**i**) is the map. Different types of structures can be seen in the centromere and chromatins. The scan-step is 12 nm.

Figure 3a is a magnified SEM image of the enclosed area in Fig. 2a. The x-ray images of the corresponding area are depicted in Fig. 3b–i. Individual chromosomes can be identified in these images. Some internal structure can be seen in the chromatin material making up the arms. We note that this does not correspond to the fibrous appearance commonly seen in SEM images^3^, which are only surface-sensitive, but is a projection throughout the entire thickness. Nevertheless, we can still observe globular structures in phase-gradient images; they exhibit high sensitivity of structural variation including the surface morphology. The appearance of transverse bands seen in the transmission image showing absorption contrast corresponds roughly to the pattern of chemically stained bands obtained by the Giemsa technique, where Giemsa or G bands (dark bands) usually are associated with differences between hetero-and eu-chromatin, which have different compositions of AT and GC bases^32^. There are also axial structures observed in the chromatids and they resemble ones observed in unstained chromosome^10^. Distributions of the Pt and Ag stain do not show a similar structure, indicating that they bind to different components. Both the absorption-contrast image (Fig. 3d) and the phase image (Fig. 3e) show a denser centromere region, but a comparable increase in Pt concentration is not observed. This may indicate that the centromere contains additional protein. It is also interesting to see some loop structures in this region from the compositional map (Fig. 3i). They are not visible in any elemental maps, possibly due to a low signal-to-noise ratio.

## Discussion

The excessively high concentration of Ag is due to silver sulfate precursor used for preparing the platinum blue stain. Its complete removal is problematic. Dense deposits of Ag contamination have been observed on the sample surface imaged using TEM^31^. Its existence also caused the change in the morphology of the chromosome prepared by the same technique. Unlike the rough surface with globules of sizes ranging in tens of nanometers that often are seen in SEM for this type of sample^3^, the sample surface observed in our investigation is much smoother and the globule size is much larger (in the range of hundreds nanometers). The x-ray images indicate that the precipitated Ag may have formed a thin film on the surface and encapsulated the chromosome sample. Because of the limited penetration power of electrons, these samples are considered to be unsuitable for a SEM study. However, this is not a problem for x-ray imaging at hard x-ray energy. Herein, we show that such a sample can still be explored effectively with hard x-rays, assuming that the excessive metal stain does not alter the sample’s internal structure. Nevertheless, a better sample preparation with minimal Ag is desirable. There are other Pt blue complexes aside from the one that we synthesized and these complexes may not require silver sulfate precursor we used in the synthesis.

The quantitative measurements of both absorption and phase give additional information about the sample. Because the real decrement of the refractive index, δ, is much larger than the imaginary component, β, the phase-contrast usually is higher than the absorption-contrast. Particularly for transparent samples, small weak-absorbing features often can only be seen by phase-contrast. In Fig 3, the phase-gradient images indeed depict the very high contrast of small features, revealing fine details about the sample. However, the resulting phase images seem blurred, showing less sharp contrast than the absorption image. There are two plausible explanations. One is because the phase image is obtained by integrating the phase gradients measured in two directions. The fundamental assumption in the differential-phase-contrast method we employed is that the phase change over the length scale of the focal spot size is linear. Therefore, as the beam penetrates through the sample, it only undergoes a small deflection effect. If the phase varies more dramatically within the beam size, the transmitted beam will not only deflect, but also change its pattern on the far-field detector. A chromosome has hierarchical structures with a length scale from hundreds of nanometers down to 2 nm (diameter of DNA). Its fine structure is well-below the beam size used for this investigation. Although our nonlinear fitting algorithm finds the best-fitting phase-gradient, the determined value is averaged over the beam size, which illuminates fine structures that cannot be resolved. The phase image, due to the integration step, suffers more from the blurring effect. In the supplementary material, this effect is illustrated using simulation data. The second cause is that the beam size, so is the resolution, is very different in two directions in this case. Because of a much smaller beam size, the horizontal phase gradient image (Fig. 3b) shows more small features than that in the vertical direction (Fig. 3c). Since the phase image is a result of integration of phase gradients in both directions, its horizontal resolution is deteriorated by the poorer resolution in the vertical direction. In other words, the resolution of the phase image (in either direction) is determined by the worse one of the phase-gradient images.

The main goal of this study is to image the distribution of Pt. Because the Pt-blue stain binds in the minor groove of the DNA with a stoichiometry of one Pt atom per 2–3 base pairs of DNA, corresponding to 1300 AMU of mass, the expected concentration of Pt is about 7 μg/cm^2^. This agrees reasonably with the measured value. At the current flux and focus size, however, the Pt signal is close to the level of the background. Therefore, it is difficult to use the Pt distribution to map DNA structure directly, but the quantitative measurements of absorption-, phase- and fluorescence-contrast images still allow us to retrieve useful structural information that is not accessible by other microscopy techniques.

In summary, we report our application of a scanning hard x-ray microscopy tool equipped with multilayer Laue lenses in studies of a human chromosome. We show that through-thickness projection images can be obtained via simultaneous absorption-, phase- and fluorescence-contrasts. X-ray images show internal structures throughout samples significantly thicker than one micron that are difficult to study using other techniques. We demonstrate that scanning hard x-ray microscopy is a very valuable tool for exploring biological samples, providing information complementary to other microscopy techniques. Despite the large thickness and unexpected high concentration of Ag stain, we can still see through the sample due to the excellent penetration power of hard x-rays. An improvement on sample preparation in future will help attain better images of chromosomes. Although in this experiment we took only a single 2D projection image, there is a potential towards 3D tomographic reconstruction by taking images at different angles in the future. X-ray nanofocusing optics are still undergoing rapid development^33^ and a nanoprobe beamline, aiming at nanometer focusing, will soon become available^34^. We believe an x-ray microscopy tool not only will bridge the gap in resolution between the optical- and electron-microscopes, but also will open new opportunities in biological studies as the result of the hard x-ray’s excellent penetration power and multi-modality imaging and quantitative analysis capabilities.

## Methods

### X-ray contrast mechanisms

When an x-ray wave propagates through a sample, it experiences amplitude attenuation and a phase retardation compared to its propagation in a vacuum due to the small difference of the refractive index of a medium from unity. At x-ray energy, the refractive index is a complex number, 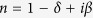, where both *δ* and *β* are very small quantities (<10^−4^ for hard x-rays). For an incident wave with unit amplitude, after passing the sample, the transmitted intensity drops down to 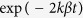 and the phase retardation introduced is *kδt*, where *k* is the wave number, 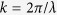, *λ* is the wavelength, and *t* is the sample thickness. The real decrement of the refractive index, *δ*, is usually much bigger than the imaginary component, *β*. Particularly for organic materials their values are three order of magnitude different. Because a detector measures intensity only, an indirect method is needed to quantify the phase change. Various phase-contrast imaging techniques have been developed^35^. Here it is done by measuring the angular shift of the nanobeam after it penetrates through the sample. The angular shift is proportional to the phase gradient introduced by the sample, and can be obtained by analyzing the translational shift of the 2D far-field image in real space^20^.

The refractive index is a function of both composition and energy. At a given energy, the attenuation factor, 2*kβt*, and the phase retardation, *kδt*, are measured at each scanned position. From a single projection-image, the thickness, *t*, is unknown. As a result, a change in attenuation factor or in phase retardation due to the thickness cannot be distinguished from that due to the composition. However, their ratio cancels *t*, and is solely a function of composition. Thus, this ratio can be used to show the variation in composition.

Fluorescence contrast is obtained by analyzing the fluorescence signals emitted from the sample. When the incident x-ray energy is above the electron-binding energy of an element, it emits characteristic fluorescence photons at specific energy. These photons are collected by a Ketek energy-dispersive detector, and analyzed by an XIA Mercury DXP to generate a spectrum. Peaks of the spectrum are indexed with the elements. The peak intensity is proportional to the concentration of the corresponding element.

### Fluorescence calibration

If we can ignore self-absorption in the sample, which is the case here, the characteristic fluorescence signal is linearly proportional to the concentration of the corresponding element. To obtain this proportionality constant, usually we measure a calibration standard with a known concentration of the element in which we are interested. Here, we use a different method to calibrate the fluorescence signal. Because the organic material is transparent to hard x-rays, the observed absorption can be assumed to be contributed solely by the metals. The absorption by a metal element is calculated directly from its concentration, which is related to the fluorescence signal with an unknown constant. Here, we attempted to seek a set of constants for individual metal elements that would result in the best agreement between the calculated absorption-contrast from the fluorescence signal, and the actual measured values. Provided that no other elements contribute appreciably to the absorption, and the correlation between fluorescence images is very weak (in other words the fluorescence image of different element looks very different), this fitting process yields a reasonable calibration on the fluorescence signal for us to obtain a quantitative measurement of the concentration.

### Sample preparation

Chromosomes were prepared from b-lymphocyte cells from a Yoruba cell line (GM18507) at passage 4, and prepared according to the protocol as described by Shemilt *et al.*^3^ and Yusuf *et al.*^9^ Briefly, cells were grown in RPMI-1640 medium (containing 20% fetal bovine serum and 1% L-Glutamine) at 37 °C in a 5% CO_2_ incubator. For synchronization, thymidine was added to each culture at a final concentration of 0.3 mg/mL, and then the sample was incubated at 37 °C for 17 hours. To obtain chromosomes at the mitotic stage, Colcemid (Invitrogen, UK) was added at a final concentration of 0.2 μg/mL for 16 hours. The chromosomes were centrifuged, re-suspended in pre-warmed hypotonic solution (0.075 M potassium chloride) for 5 minutes and prepared after being fixed in three changes of 3:1 methanol acetic acid. We prepared mounted chromosome samples for X-ray imaging according to a protocol published by Nishino *et al.*^10^ with slight modifications. Briefly, the chromosomes were fixed for 10 minutes in 0.5% glutaraldehyde containing 10 mM Hepes-KOH, and 5 mM MgCl_2_, then placed on a 100-nm-thick silicon nitride membrane “window”. The chromosomes were allowed to stick to the membrane’s surface and then were washed with water and stained with 150 μM of SYBR Gold (to permit optical fluorescence imaging). After washing the sample in water, its status was validated under a Zeiss Axio Z2 microscope with Isis software. While still bound to the membrane, the chromosomes then were stained with 5 mM platinum blue (Pt(CH_3_CONH)_2_) prepared according to Wanner and Formanek^26^ for 30 minutes and washed in water for 15 minutes before X-ray imaging. The synthesis of the platinum blue complex is the following. Potassium tetrachloroplatinate (2 g) was mixed with acetonitrile (3 mL) in 40 mL of water at room temperature. The reaction was left for 10 days after which yellow crystals were obtained. The liquid was decanted, and the crystals were air-dried and weighed. The crystals were then mixed vigorously with the same amount of silver sulfate (Ag_2_SO_4_) in a fivefold volume of water until the blue color reached its maximum intensity after which a 10-fold volume of methanol was added to the solution. The solution was filtered followed by the addition of diethyl ether to the filtrate to precipitate the platinum blue. The platinum blue was then filtered out from the solution and air-dried. As a powder, or in water, platinum blue is stable for at least 1 month at room temperature.

## Additional Information

**How to cite this article**: Yan, H. *et al.* Multimodality hard-x-ray imaging of a chromosome with nanoscale spatial resolution. *Sci. Rep.* **6**, 20112; doi: 10.1038/srep20112 (2016).

## Supplementary Material

Supplementary video

Supplementary Information

## References

[b1] Woodcock C. L. & Ghosh R. P. Chromatin Higher-order Structure and Dynamics. CSH Perspect. Biol. 2, a000596 (2010). 10.1101/cshperspect.a000596 PMC2857170 20452954

[b2] Matsuda A. *et al.* Condensed Mitotic Chromosome Structure at Nanometer Resolution Using PALM and EGFP- Histones. PLoS ONE 5, e12768 (2010). 20856676 10.1371/journal.pone.0012768 PMC2939896

[b3] Shemilt L. A., Estandarte A. K. C., Yusuf M. & Robinson I. K. Scanning electron microscope studies of human metaphase chromosomes. Philos. Trans. R. Soc. A-Math. Phys. Eng. Sci. 372, 20130144 (2014). 10.1098/rsta.2013.0144 PMC3900039 24470422

[b4] Henderson R. The potential and limitations of neutrons, electrons and X-rays for atomic resolution microscopy of unstained biological molecules. Q. Rev. Biophys. 28, 171–193 (1995). 7568675 10.1017/s003358350000305x

[b5] Ris H. Stereoscopic Electron Microscopy of Chromosomes in Methods Cell Biol. Vol. 22 (ed Turner James N.) 77–96 (Academic Press, 1981). 7022124 10.1016/s0091-679x(08)61871-3

[b6] Engelhardt P. Electron Tomography of Chromosome Structure in Encyclopedia Anal. Chem. (John Wiley & Sons, Ltd, 2006).

[b7] Harauz G., Borland L., Bahr G., Zeitler E. & van Heel M. Three-dimensional reconstruction of a human metaphase chromosome from electron micrographs. Chromosoma 95, 366–374 (1987). 3652820 10.1007/BF00293184

[b8] Schroeder-Reiter E., Pérez-Willard F., Zeile U. & Wanner G. Focused ion beam (FIB) combined with high resolution scanning electron microscopy: A promising tool for 3D analysis of chromosome architecture. J. Struct. Biol. 165, 97–106 (2009). 19059341 10.1016/j.jsb.2008.10.002

[b9] Yusuf M. *et al.* Staining and Embedding of Human Chromosomes for 3-D Serial Block-Face Scanning Electron Microscopy. Biotechniques 57, 302–307 (2014). 25495730 10.2144/000114236

[b10] Nishino Y., Takahashi Y., Imamoto N., Ishikawa T. & Maeshima K. Three-Dimensional Visualization of a Human Chromosome Using Coherent X-Ray Diffraction. Phys. Rev. Lett. 102, 018101 (2009). 19257243 10.1103/PhysRevLett.102.018101

[b11] Chen S. *et al.* The Bionanoprobe: hard X-ray fluorescence nanoprobe with cryogenic capabilities. J. Synchrotron Radiat. 21, 66–75 (2014). 24365918 10.1107/S1600577513029676 PMC3874019

[b12] De Samber B. *et al.* Hard X-ray nanoprobe investigations of the subtissue metal distributions within Daphnia magna. Anal. Bioanal. Chem. 405, 6061–6068 (2013). 23681201 10.1007/s00216-013-7019-6

[b13] Yuan Y. *et al.* Epidermal Growth Factor Receptor Targeted Nuclear Delivery and High-Resolution Whole Cell X-ray Imaging of Fe3O4@TiO2 Nanoparticles in Cancer Cells. ACS Nano 7, 10502–10517 (2013). 24219664 10.1021/nn4033294 PMC3919441

[b14] Andrews J. C., Meirer F., Liu Y., Mester Z. & Pianetta P. Transmission X-ray microscopy for full-field nano imaging of biomaterials. Microsc. Res. Techniq. 74, 671–681 (2011). 10.1002/jemt.20907 PMC2992572 20734414

[b15] Jiang H. *et al.* Nanoscale Imaging of Mineral Crystals inside Biological Composite Materials Using X-Ray Diffraction Microscopy. Phys. Rev. Lett. 100, 038103 (2008). 18233041 10.1103/PhysRevLett.100.038103

[b16] Nazaretski E. *et al.* Pushing the limits: an instrument for hard X-ray imaging below 20 nm. J. Synchrotron Radiat. 22, 336–341 (2015). 25723934 10.1107/S1600577514025715

[b17] Yan H. F., Conley R., Bouet N. & Chu Y. S. Hard x-ray nanofocusing by multilayer Laue lenses. J. Phys. D: Appl. Phys. 47, 263001 (2014).

[b18] Yan H. F. *et al.* Takagi-Taupin description of x-ray dynamical diffraction from diffractive optics with large numerical aperture. Phys. Rev. B 76, 115438–115413 (2007).

[b19] Huang X. *et al.* 11 nm hard X-ray focus from a large-aperture multilayer Laue lens. Sci. Rep. 3, 3562 (2013). 24356395 10.1038/srep03562 PMC3868962

[b20] Kang H. C. *et al.* Oxidation of PtNi nanoparticles studied by a scanning X-ray fluorescence microscope with multi-layer Laue lenses. Nanoscale 5, 7184–7187 (2013). 23674261 10.1039/c3nr00396e

[b21] Yan H. *et al.* Quantitative x-ray phase imaging at the nanoscale by multilayer Laue lenses. Sci. Rep. 3, 1307 (2013). 23419650 10.1038/srep01307 PMC3575587

[b22] Yan H. & Chu Y. S. Optimization of multilayer Laue lenses for a scanning X-ray microscope. J. Synchrotron Radiat. 20, 89–97 (2013). 23254660 10.1107/S0909049512044883

[b23] Bahr G. F. & Golomb H. M. Karyotyping of Single Human Chromosomes from Dry Mass Determined by Electron Microscopy. Proc. Natl. Acad. Sci. 68, 726–730 (1971). 5279514 10.1073/pnas.68.4.726 PMC389029

[b24] Williams S. *et al.* Measurements of wet metaphase chromosomes in the scanning transmission X-ray microscope. J. Microsc. 170, 155–165 (1993).

[b25] Shemilt L. *et al.* Karyotyping Human Chromosomes by Optical and X-Ray Ptychography Methods. Biophys. J. 108, 706–713 (2015). 25650937 10.1016/j.bpj.2014.11.3456 PMC4317545

[b26] Wanner G. & Formanek H. Imaging of dna in human and plant chromosomes by high-resolution scanning electron-microscopy. Chromosome Res. 3, 368–374 (1995). 7551552 10.1007/BF00710018

[b27] da Silva J. C. *et al.* Mass Density and Water Content of Saturated Never-Dried Calcium Silicate Hydrates. Langmuir 31, 3779–3783 (2015). 25794183 10.1021/la504478j

[b28] Shapiro D. A. *et al.* Chemical composition mapping with nanometre resolution by soft X-ray microscopy. Nat. Photonics 8, 765–769 (2014).

[b29] Jones M. W. M. *et al.* Mapping biological composition through quantitative phase and absorption X-ray ptychography. Sci. Rep. 4, 6796 (2014). 25348877 10.1038/srep06796 PMC4210942

[b30] Brown D. B., Burbank R. D. & Robin M. B. Platinblau [blue platinum amide complex]. J. Am. Chem. Soc. 91, 2895–2902 (1969).

[b31] Inaga S. *et al.* Platinum blue as an alternative to uranyl acetate for staining in transmission electron microscopy. Arch. Histol. Cytol. 70 (2007). 10.1679/aohc.70.43 17558143

[b32] Sumner A. T. The nature and mechanisms of chromosome banding. Cancer Genet. Cytogen. 6, 59–87 (1982). 10.1016/0165-4608(82)90022-x 7049353

[b33] Ice G. E., Budai J. D. & Pang J. W. L. The Race to X-ray Microbeam and Nanobeam Science. Science 334, 1234–1239 (2011). 22144618 10.1126/science.1202366

[b34] Chu Y. S. *et al.* Hard X-ray Nanoprobe. http://www.bnl.gov/ps/beamlines/beamline.php?b=HXN (2014) (Date of access: 19/06/2015).

[b35] Liu Y., Nelson J., Holzner C., Andrews J. C. & Pianetta P. Recent advances in synchrotron-based hard x-ray phase contrast imaging. J. Phys. D: Appl. Phys. 46, 494001 (2013).

