# Supplementary material for: Multimodality hard-x-ray imaging of a chromosome with nanoscale spatial resolution
Source: Sci Rep. 2016 Feb 5;6:20112. doi: 10.1038/srep20112 (PMC4742846; doi:10.1038/srep20112)
Supplement: Supplementary Information [file srep20112-s2.pdf]

Supplementary Information

Multimodality hard-x-ray imaging of a chromosome with nanoscale spatial resolution

Hanfei Yan[1*], Evgeny Nazaretski[1], Kenneth Lauer[1], Xiaojing Huang[1], Ulrich Wagner[2], Christoph Rau[2], Mohammed Yusuf[3,4], Ian Robinson[3,4], Sebastian Kalbfleisch[1], Li Li[1], Nathalie Bouet[1], Juan Zhou[1], Ray Conley[1,5], and Yong S. Chu[1]

1. National Synchrotron Light Source II, Brookhaven National Laboratory, Upton, NY 11973, USA
2. Diamond Light Source Ltd, Didcot, Oxfordshire, OX11 0DE, UK
3. London Centre for Nanotechnology, University College London, London, WC1H 0AH, UK
4. Research Complex at Harwell, Rutherford Appleton Laboratory, Didcot, OX11 0FA, UK
5. Advanced Photon Source, Argonne National Laboratory, Argonne, Illinois 60439, USA

* Correspondence and requests for materials should be addressed to H. Y. (hyan@bnl.gov)

Blurring effect in the phase image

In this study the phase image looks more blurred than the transmission image. In order to fully understand this phenomenon, we conducted a theoretical study using simulation data. Fig. S1a is the original image of the amplitude and the phase variations of the object. For a fair comparison, its amplitude and phase variations are the same. The image size is 130 x 162 pixels. The object is raster-scanned by a focused beam from a zone plate, with a full-width-of-half-maximum (FWHM) beam size of 4 pixels. The scan step is one pixel, and a total of 130 x162 data points are taken, covering the whole image. Figs. S1b and S1c are the recovered amplitude and phase images using DPC, respectively. Ideally, both of them should be identical to the original image, Fig. S1a. Due to the large beam size, however, both images are blurred, particularly the phase. This agrees with what we observed in this experiment.

For a better understanding, we show in Figs. S2a and S2b the far-field image recorded on the downstream detector when the object is absent and present in the nanobeam, respectively. Both depict a typical ring pattern for a Fresnel zone plate, but the intensity distribution is more uniform in Fig. S2a (without sample) than that in Fig. S2b (with sample). The reason is that the complex transmission function of the object varies significantly within the beam size in this case; the assumption that locally the phase varies linearly is no longer sufficient to describe the object. When the altered wavefront of the nanobeam propagates to the downstream detector, it undergoes not only a deflection in direction but also a pattern change. The latter is not captured in the employed differential phase algorithm, which attempts to find the overall shift of the whole pattern on the detector only, neglecting how the pattern

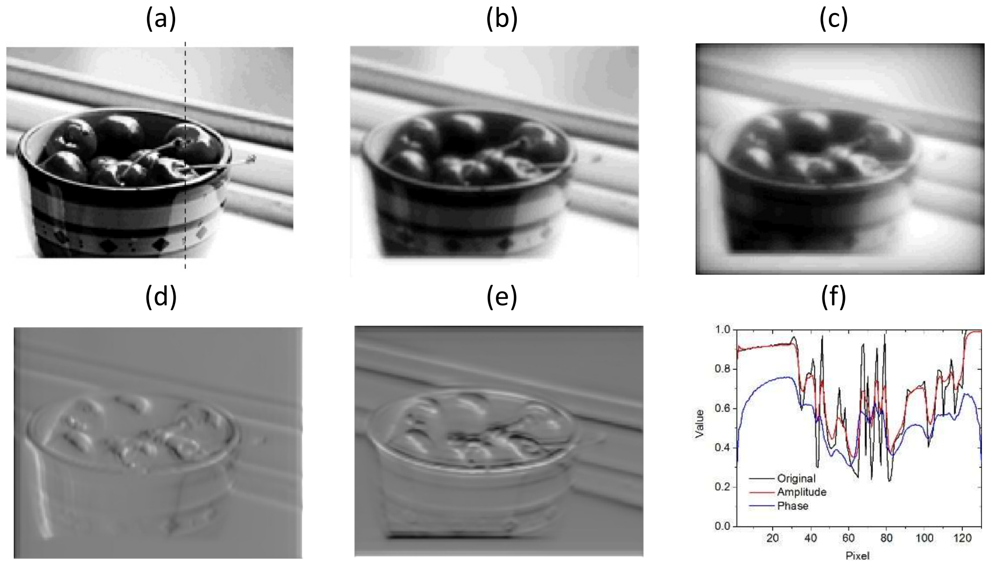

Figure S1. The original picture (a), the amplitude (b) and phase (c) images. Ideally, (b) and (c) should be identical to (a). (d) and (e) are phase-gradient images in horizontal and vertical directions, respectively. (f) is a comparison of line profiles of the original, the amplitude and the phase at the same position. Note: there is ab arbitrary offset for the phase. The line is shown in (a). Evidently phase is blurred more significantly.

itself varies. As a result, the calculated phase-gradient is an averaged value over the illumination area and the corresponding image will be blurred because of a large beam size. In Figs. S1d and S1e, we plot the phase-gradient images in horizontal and vertical directions, respectively. They show enhanced contrast at sharp boundaries, but in general the resolution is not better than the amplitude image. The phase, because it is obtained by integrating two already-blurred phase-gradients, is affected most significantly. In Fig. S1f line profiles across the same location in the original, the reconstructed amplitude and phase images are compared. The smearing effect in the latter two is clearly illustrated.

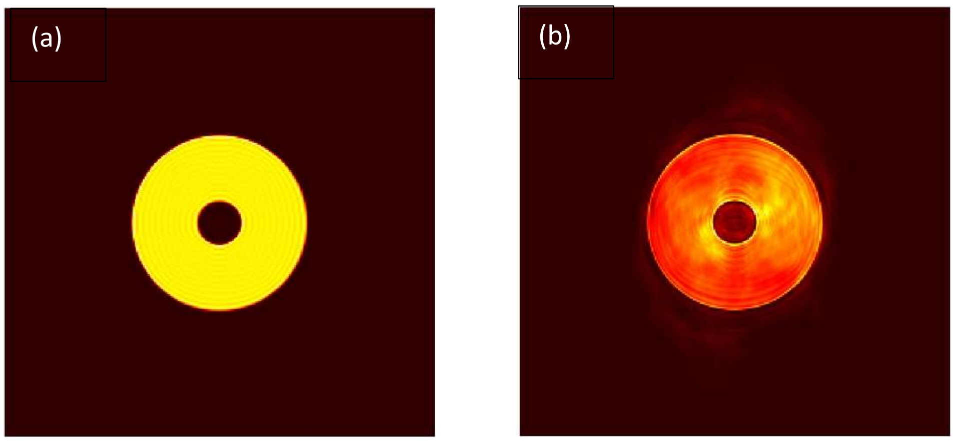

Figure S2. The far-field pattern taken without (a) and with (b) the sample. Fine pattern changes can be seen in (b).

It is important to note that the phase images shown in Figs. 2 and 3 are not as featureless as they look like, although there are blurring effects due to the technique itself. Because the organic material introduces a lot of low-frequency variations in the phase image, small features become less apparent. If we apply a high-pass filter to the phase images, they show a lot more small details (Fig. S3).

(a) (b)

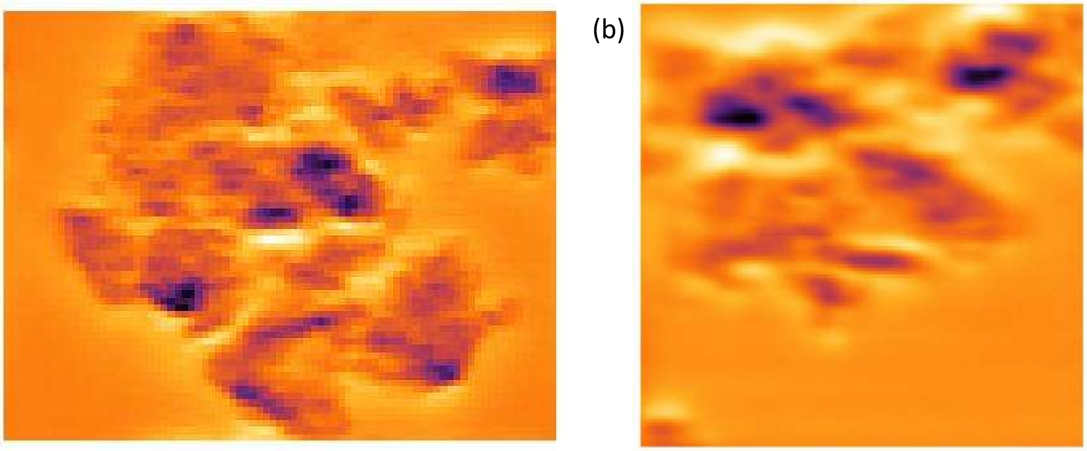

Figure S3. Phase images after applying a high-pass filter to suppress the low-frequency variation. (a) corresponds to Fig. 2e and (b) to Fig. 3e.

Focus measurements

Two different methods were used to measure the image resolution. One is the conventional knife-edge type scan based on the fluorescence signal. The scanned object is a double-line test pattern made of Pt. It is a high-aspect ratio structure with a line/gap width of 20 nm and a height of 200 nm. When it is scanned, the yielded Pt fluorescence signal is a convolution of the object function and the nanobeam intensity profile. Assuming a perfect structure and a perfect angular alignment of the side-wall to the incident nanobeam, we can consider the object function to be two step functions. We further assume the nanobeam intensity profile can be described by a Gaussian function. Using a nonlinear fitting algorithm a best fit to the experimental data is achieved. This yields a full-width-of-half-maximum (FWHM) beam size of 19 (H) x 41 (V) nm^2, shown in Fig. S4a and S4b. One shall note that this value is the upper bound of the actual beam size, since it includes all the broadening effects due to the imperfection of the test-pattern, misalignment of the side-wall to the beam, and vibration.

Ptychography was also employed to characterize the beam size. Different from our previous work where a grating structure was used to characterize the focus profile from a single MLL [1], in this case the object was a spike pattern with a 2D structure. It was scanned following a spiral pattern with sufficient overlapping for optimum reconstruction performance [2]. A series of far-field images were recorded at each scanned position. Iterative ptychography algorithm were then applied to reconstruct both the probe and the object simultaneously. More experimental details can be found elsewhere [3]. In addition to the benefit of obtaining the phase information, this method decouples the probe and the object completely, therefore yielding a more accurate measure of the probe size. In Figure S5, we show both

the intensity distribution of the reconstructed probe on the focal plane and the object. As is evident from the object image, the quality of the reconstruction is very high. The probe size, measured along central lines, is 13 × 33 nm^2. As expected, this number is smaller than that obtained from knife-edge scans, since ptychography removes the broadening effects from the imperfection of the test structure and the misalignment.

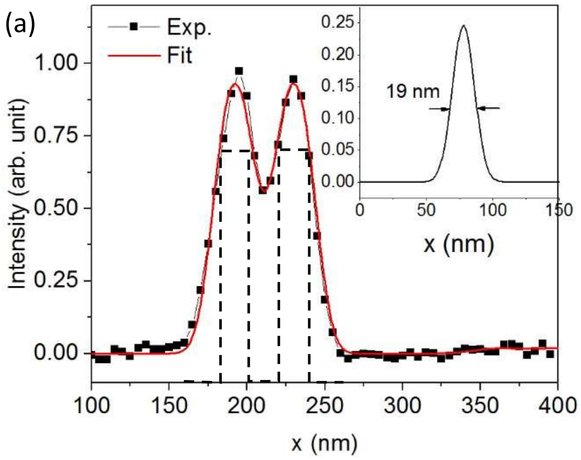

Figure S4. Scan line profiles in x a) and y b) directions. They are assumed to be the convolution of the object function (dashed line) and the nanobeam (insets). The beam size (FWHM) is 19 × 41 nm^2.

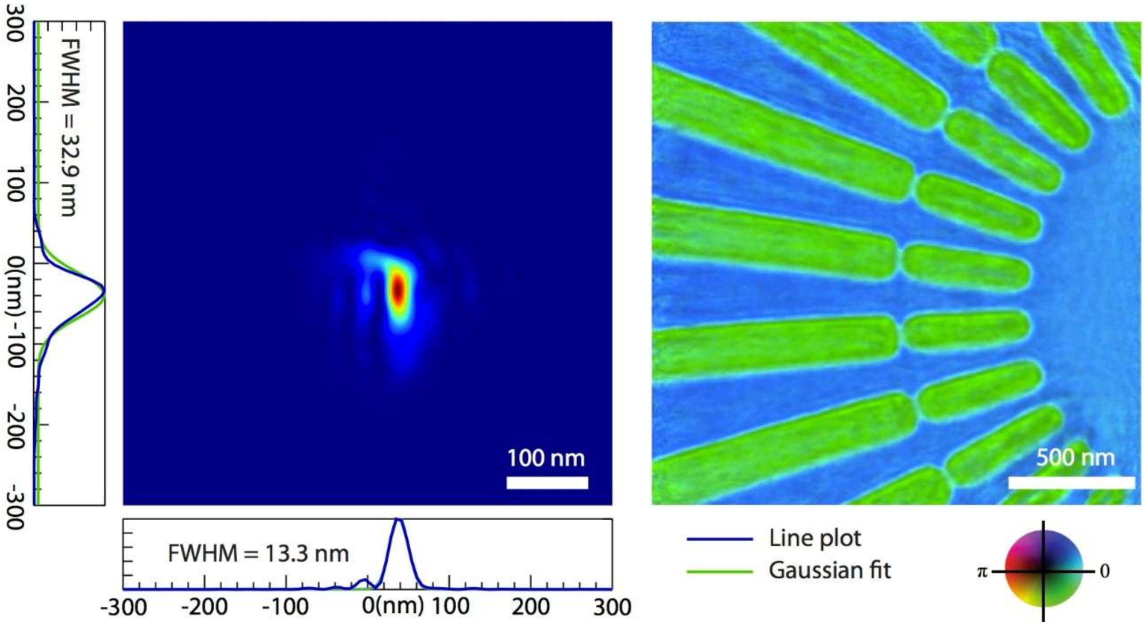

Figure S5. Reconstructed beam intensity distribution on the focal plan a) and the test pattern b). Line profiles across the center are also shown in a). The beam size (FWHM) obtained from this technique is 13 × 33 nm^2, smaller than that measured from knife-edge scans.

Image resolution

In addition to point spread function, another common method of evaluating image resolution is to use power spectral density (PSD) analysis. In general, the power density of an image decreases as the frequency increases. At high-frequency, the power density fluctuation is solely due to background noise and is not related to real spatial variation. Therefore, one can define a cut-off frequency beyond which the noise is dominant and the spatial frequency is not resolvable. The half period of the cut-off frequency is then the spatial resolution of the image. In Fig. S6 we plot the corresponding PSD of the transmission, phase-gradients and phase images shown in Fig. 3 in the text. Although the cut-off frequency is slightly different from image to image, the resolving power is no worse than 15 μm^{-1} in x and 20 μm^{-1} in y, corresponding to spatial resolution of 33 nm and 25 nm, respectively. This serves as a bottom limit of the resolution using this method. Because the relatively large pixel size (12 nm) and relatively small image size (126 x 126), the cut-off frequency has a relatively large uncertainty and we expect that this method will yield less accurate resolution number than the point spread function.

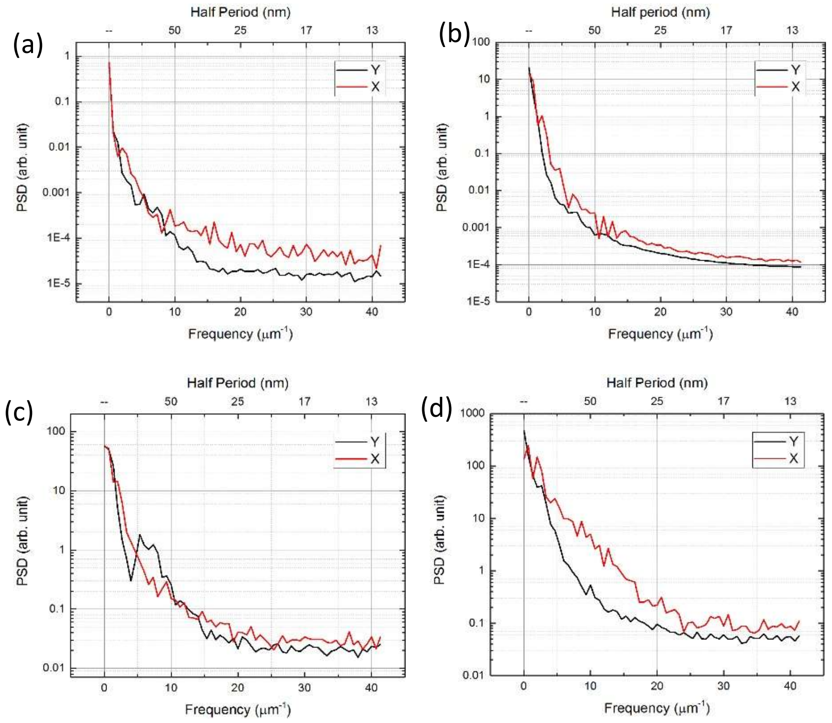

Figure S6. Power spectral density variations of transmission a), phase b), phase-gradient x c) and phase-gradient y d) images. The cut-off frequency is slightly different, depending on the type of images. A bottom bound of the resolving power is around 20 x 15 μm^{-1}, consistent with the point spread function analysis.

Optical and fluorescence imaging

Optical fluorescence images of the Pt stained sample were taken prior to x-ray imaging (Figure S7). Stained chromosomes can be observed clearly on the silicon nitride window. At the x-ray examined spot, however, the fluorescence is faint. This may be due to the fact that there is excessive Ag deposition.

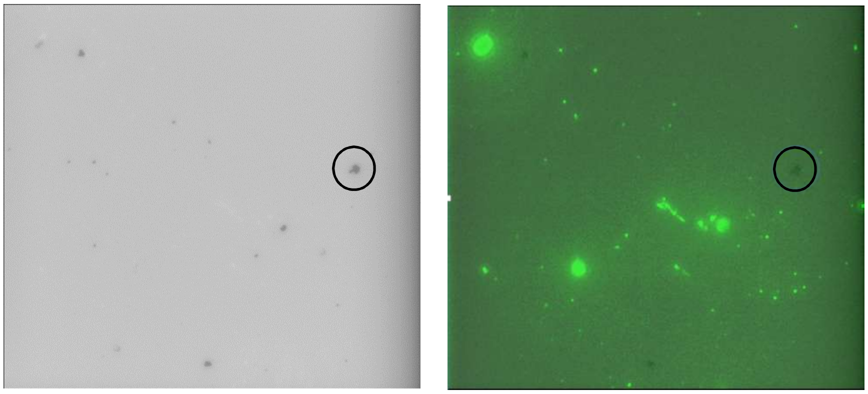

Figure S7. (a) Optical image of the sample prior to x-ray imaging. (b) Fluorescence image overlaid with the optical one. The circled area is x-ray examined. Very faint fluorescence can be observed from the Pt stain, which may be due to the excessive Ag deposition.

References

1 Huang, X. *et al.* 11 nm hard X-ray focus from a large-aperture multilayer Laue lens. *Scientific Reports* **3**, 3562, doi:10.1038/srep03562 (2013).

2 Huang, X. *et al.* Optimization of overlap uniformness for ptychography. *Optics Express* **22**, 12634-12644, doi:10.1364/oe.22.012634 (2014).

3 Nazaretski, E. *et al.* Pushing the limits: an instrument for hard X-ray imaging below 20 nm. *Journal of Synchrotron Radiation* **22**, 336-341, doi:doi:10.1107/S1600577514025715 (2015).

Video caption

The video shows the variation of the far-field diffraction pattern as the nanobeam scans over the chromosome sample. One frame corresponds to one data point.
